# Diagnostic efficacy of pulse oximetry versus Ankle-Brachial index for peripheral vascular disease in type 2 diabetes

**DOI:** 10.6026/973206300223496

**Published:** 2026-06-30

**Authors:** Mohammad Kamran Iqbal, Jayballabh Kumar, Ritu Adhana, Vinod Kumar Singh

**Affiliations:** 1 Department of Physiology Teerthanker Mahaveer Medical College and Research Center, Moradabad, Uttar Pradesh, India; 2 Department of General Medicine Teerthanker Mahaveer Medical College and Research Center, Moradabad, Uttar Pradesh, India

**Keywords:** Peripheral vascular disease (PVD), type 2 diabetes mellitus (T2DM), ankle-brachial index (ABI), pulse oximetry, diagnostic accuracy

## Abstract

The Peripheral Vascular Disease (PVD) is a debilitating macrovascular complication associated with Type 2 Diabetes Mellitus (T2DM) with significant mortality. This remains undiagnosed in the asymptomatic cases due to diabetic neuropathy. Therefore, it is of interest to evaluate the diagnostic accuracy of the pulse oximetry against the gold-standard Ankle-Brachial Index (ABI). Data shows a high prevalence of PVD with ABI, whereas the pulse oximetry validated moderate sensitivity but low specificity, limiting its utility as a standalone diagnostic tool. While ABI remains the superior standard for definitive diagnosis, pulse oximetry offers a viable, cost-effective preliminary screening option for high-risk populations in resource-constrained healthcare centers.

## Background:

The global healthcare system faces a profound epidemiological shift. It is marked by a transition from communicable diseases to the rising prevalence of non-communicable diseases (NCDs), driven by rapid urbanization and digitalization [2]. Among NCDs, diabetes mellitus has emerged as a global public health emergency. Its incidence rate is soaring despite international prevention efforts [5]. Low- and Middle-Income Countries (LMICs) bear a disproportionate burden, leading to a multi-fold increase in diabetes prevalence, premature mortality and disability. In South-East Asia alone, approximately 90.2 million people currently live with diabetes. However, this number is expected to rise to 151.5 million by 2045 [1]. Type 2 Diabetes Mellitus (T2DM) is a chronic metabolic disorder characterized by persistent hyperglycemia due to insulin resistance or inadequate insulin production [3]. The risk factors associated with T2DM include overweight, physical inactivity, and poor eating habits, and these can be modified, whereas non-modifiable factors are genetic susceptibility and ethnicity. A major challenge is the "rule of halves", where about half of the adult diabetes cases remain undiagnosed [4]. India ranks third in the world for the number of unidentified diabetes cases [6]. The delayed diagnosis leads to severe complications, including microvascular issues (retinopathy, nephropathy and neuropathy) and macrovascular (coronary heart disease, stroke, and peripheral vascular disease) pathologies [9]. Peripheral Vascular Disease (PVD), a major macrovascular complication, develops at higher rates in diabetic patients than in non-diabetic individuals [7]. The PVD is marked by progressive blockage of peripheral arteries, causing reduced blood flow to the limbs and limb ischemia. Hyperglycemia causes advanced glycation end products (AGEs) and reactive oxygen species (ROS), which damage the endothelium and promote autoimmune inflammation [3]. The macrophages release pro-inflammatory cytokines like TNF-α and IL-6, leading to vascular damage [8]. The PVD usually goes undiagnosed until advanced stages, as the early symptoms are vague and co-existing peripheral neuropathy can mask various signs like intermittent claudication or lower extremity pain [7]. As a result, people with serious problems such as non- healing ulcers or gangrene may require limb amputation. The Ankle-Brachial Index (ABI) [10] is a standard and widely used non-invasive method to find peripheral vascular disease (PVD) and shows high sensitivity and specificity (90-95%). If the ABI value is below 0.9, then it means there is PVD. However, ABI measurement is not easy as it requires Doppler equipment, trained personnel, and processes that take a long time. The ABI may not work as well in elderly people with diabetes due to medial artery calcification, which gives falsely elevated readings [14]. Considering these limitations, an alternate tool, pulse oximetry, which measures peripheral blood oxygen saturation (SpO2), through spectrophotometry, is proposed as a feasible alternative. It is a non-invasive, cost- effective, and requires minimal training, making it suitable for primary care [11]. The pulse oximetry may theoretically indicate vascular obstruction by identifying reductions in oxygen saturation in the extremities [12]. Many studies confirmed that pulse oximetry has specificity, but there is conflicting information about its sensitivity as compared to ABI [13, 15, 16, 17]. Therefore, it is of interest to evaluate the diagnostic accuracy of pulse oximetry compared to ABI in identifying PVD in T2DM patients.

## Materials and Methods:

## Study design and setting:

The cross-sectional study was conducted in the hospital-based setting. It was carried out in the Outpatient Department (OPD), Department of Medicine, at Teerthanker Mahaveer Medical College and Research Center, Moradabad, Uttar Pradesh, India. The duration of the study was 16 months and concluded in October 2025.

## Sampling strategy and sample size:

The participants were selected using a purposive sampling technique. The sample size was calculated using the formula and the calculation yielded a statistically significant sample size of 121 participants.

## Inclusion and exclusion criteria:

The subjects included in the study were adults aged between 35 and 60 years who had been diagnosed with T2DM for a duration of 5 years or more, regardless of their glycemic control or treatment status. Exclusion criteria included patients with a history of hypercoagulable states, congestive heart failure, suspected arteritis, vascular diseases, or an inability to lie in the supine position. Furthermore, people with a history of smoking, alcohol use, or substance addiction were eliminated to ensure that diabetes was the sole risk factor for vascular pathology.

## Data collection procedure:

A written informed consent was obtained prior to the patients' enrollment in the study. Demographic and clinical data were collected using a structured proforma.

## Pulse oximetry: 

The values were measured utilizing a finger pulse oximeter (Hesley Inc. PC-60B1). The patient was positioned in a supine position on an examination table. The SpO_2_ values were obtained simultaneously from the right index finger, left index finger, right big toe, and left big toe. Thereafter, each lower leg was raised 12 inches above the table surface (heel to table), and SpO_2_ values were measured for the big toes. The PVD was characterized by a decrease above 2% in big toe SpO_2_ relative to finger SpO_2_, or a reduction greater than 2% in big toe SpO_2_ following limb elevation.

## Ankle-Brachial index (ABI): 

Ankle-Brachial Index (ABI) was measured using a sphygmomanometer (Diamond Dial Deluxe BP device) and a handheld vascular Doppler equipped with an 8 MHz probe (EMCO D580). The systolic blood pressure was measured at the brachial arteries (right and left) and the ankle arteries ( dorsalis pedis and posterior tibial). The greater of the two ankle pressures for each leg was divided by the greater of the two brachial pressures to compute the ABI [10].

## Statistical analysis:

The SPSS version 27 statistical software was used for data analysis. The quantitative factors, such as age, duration of diabetes, SpO_2_ levels, and ABI values, were presented as mean ± standard deviation (SD). The qualitative characteristics, such as gender and the prevalence of comorbidities, were expressed as frequencies and percentages. The diagnostic accuracy was evaluated by cross-tabulating pulse oximetry results with ABI (the gold standard). In the study, we used formulas for diagnostic accuracy to figure out sensitivity, specificity, Positive Predictive Value (PPV), Negative Predictive Value (NPV), and overall accuracy. A p-value < 0.05 was considered statistically significant.

## Results:

As shown in Table 1, the study population comprised 121 diabetic patients having a mean age of 54.95 ± 7.02 years. There was a slight male preponderance, with 55.4% (n=67) males and 44.6% (n=54) females. The mean duration of diabetes was 10.52 ± 6.11 years. A significant proportion of participants (47.1%) had disease duration of 5-8 years, while 32.2% had duration of 9-12 years. Comorbidities were present in 50.4% (n=61) of the cohort. Among those with comorbidities, 16.5% had been managing these conditions for over 15 years, indicating a substantial chronic disease burden. The pulse oximetry values revealed distinct physiological differences between upper and lower extremities. The mean SpO^2^ in the right index finger was 98.89 ± 1.41%, compared to 96.68 ± 2.52% in the right big toe (in supine position). Upon elevation, the right big toe SpO2 dropped to 95.53 ± 2.71%. The mean percentage difference between the right index finger and right big toe was 2.21 ± 2.36%. Based on pulse oximetry criteria (finger-toe difference), 57.9% and 61.2% of participants were classified as PVD positive in right and left limbs, respectively. Using the supine limb-elevation, 55. 4% were classified as PVD positive in the right limb and 63.6% in the left limb. The average ABI values were 0.76 ± 0.16 for the right limb and 0.77 ± 0.15 for the left limb, signifying a significant prevalence of vascular impairment in the study group. Utilizing the conventional diagnostic threshold of ABI less than 0.9, peripheral vascular disease (PVD) was identified in 86.8% (n=105) of participants in the right limb and 89.3% (n=108) in the left leg. The inter-limb comparison of ABI demonstrated significant consistency, achieving an accuracy of 94.21% in the assessment of right and left ABI diagnoses. Cross-tabulation of pulse oximetry results against ABI revealed significant limitations in the former's diagnostic performance. In a comparative analysis evaluating diagnostic trends for the right limb, pulse oximetry based on elevation difference was assessed against the Ankle-Brachial Index (ABI) as the reference standard. The results yielded 47 true positive and 7 true negative cases. Furthermore, the diagnostic data revealed 20 false positives and 58 false negatives. The calculated sensitivity was 86.56% when comparing pulse oximetry against ABI, but the specificity was notably low at 12.96%. However, when analyzing the pulse oximetry metrics internally (Index vs. Toe difference), sensitivity was approximately 62.68% and specificity was 48.14%. For the left limb, pulse oximetry had a sensitivity of 92.2% against ABI of only 15.9% as specificity. However, the diagnostic accuracy of pulse oximetry was found to be between 53% and 64%, driven largely by a high rate of false positives and a failure to specifically identify disease-free individuals compared to the robust discrimination of ABI.

## Discussion:

The study highlights a disturbingly high prevalence of PVD (~88%) among diabetic patients attending a tertiary care center in North India. This figure significantly surpasses prevalence rates documented in Western literature and several urban Indian researches, including the 3.2% prevalence noted in the Chennai Urban Population Study. This gap is likely due to the inclusion of individuals with chronic diabetes (mean duration >10 years) and substantial comorbidities, indicating a population at an advanced stage of vascular development. The elevated prevalence corresponds with the finding that the risk of PVD increases with age and the duration of diabetes, since sustained hyperglycemia promotes chronic inflammation and endothelial injury [1]. The main aim was to evaluate the utility of pulse oximetry as a low-cost alternative to ABI. The results indicated that while pulse oximetry possesses moderate sensitivity (~~63%), it suffers from poor specificity (43-48%). This finding contrasts with earlier studies by Parameswaran *et al.* [18], who reported a specificity of 97%, and Kwon and Lee [19], who reported 96.1%. Nevertheless, our findings are more consistent with the research conducted by Shetty *et al.* (2025), who reported diminished diagnostic accuracy for pulse oximetry [15]. The diminished specificity in our sample may result from physiological factors such as peripheral neuropathy, which can modify local blood flow regulation, and changes in skin pigmentation that influence spectrophotometric measurements. Likewise, the 12-inch limb elevation test depends on gravitational forces to provoke ischemia; in patients with significant collateral circulation or mixed venous pathology, variations in oxygen saturation may not occur in a linear fashion, resulting in erroneous classifications. The combination of ABI and pulse oximetry has been suggested to improve diagnostic yield. Siao *et al.* (2018) [20] reported that a combined approach increased sensitivity to 88.1%. In our study, adding pulse oximetry to ABI did not significantly enhance overall accuracy. The high false- positive rate of pulse oximetry tends to dilute the precision of ABI, suggesting that pulse oximetry should not be used as a confirmatory tool. Instead, its value lies in its high negative predictive value in some contexts or as a "rule-in" screening tool in primary health care settings where Doppler equipment is unavailable [11]. If a patient tests positive on pulse oximetry, they should be referred for definitive ABI testing. The limitations of this study were that it was a hospital-based design, which may have caused a selection bias towards more severe cases, and a relatively small sample size (n=121). Additionally, the study did not assess arterial calcification via other tools like toe-brachial index (TBI), which could have influenced ABI readings in elderly patients [14]. These mechanism-driven screening can serve as a beneficial tool in advanced management of this complication, thereby improving the disease outcomes [21]. In the future, multicentric studies may be conducted with larger cohorts to validate these findings across a diverse population.

## Conclusion:

We show that ABI remains the superior and most reliable non-invasive diagnostic standard for PVD in T2DM. Pulse oximetry offers a practical, albeit less specific, screening alternative for resource-limiting environments. This could facilitate earlier detection in rural areas, potentially reducing the burden of disease through early care and management.

## Ethical approval:

The study was approved by the Teerthanker Mahaveer University's Institutional Ethics Committee via ref no. TMU/IEC/2024-25/PG/88 dated 10-06-2024.

## Funding:

The study was funded for financial support from the Department of Health Research (DHR) for the MD/MS/DM/MCh/DNB/DrNB/MDS Thesis Program via letter no. HRD/DHR-ICMR/PG-2024/0282 dated 24-01-2025.

## Figures and Tables

**Table 1:** Comprehensive summary of demographic, clinical, hemodynamic, and diagnostic performance data in study participants (N = 121)

**Parameter**	**Findings**
A. Demographic and Clinical Characteristics	
Age (years), Mean ±SD	54.95 ±7.02
Duration of Diabetes (years), Mean ±SD	10.52 ±6.11
Gender Distribution	Male: 67 (55.4%); Female: 54 (44.6%)
Diabetes Duration Distribution	5-8 years: 57 (47.1%); 9-12 years: 39 (32.2%); 13-16 years: 9 (7.4%); 17-20 years: 6 (5.0%);>20 years: 10 (8.3%)
Comorbidity Status	Present: 61 (50.4%); Absent: 60 (49.6%)
Comorbidity Duration	<5 years: 9 (7.4%); 5-10 years: 18 (14.9%); 11-15 years: 14 (11.6%); >15 years: 20 (16.5%); No comorbidity: 60 (49.6%)
B. Pulse Oximetry (Mean ±SD)	
Right Index Finger SpO_2_ (%)	98.89 ±1.41
Left Index Finger SpO_2_ (%)	99.02 ±1.12
Right Great Toe SpO_2_ (%)	96.68 ±2.52
Right Great Toe (12-inch elevation) SpO_2_ (%)	95.53 ±2.71
Left Great Toe SpO_2_ (%)	97.02 ±2.33
Left Great Toe (12-inch elevation) SpO_2_ (%)	95.63 ±2.64
% Difference: Right Index vs Right Toe	2.21 ±2.36
% Difference: Right Toe vs Elevated Right Toe	1.15 ±2.80
% Difference: Left Index vs Left Toe	2.01 ±2.19
% Difference: Left Toe vs Elevated Left Toe	1.39 ±2.63
C. PVD Classification by Pulse Oximetry	
Right Index-Toe Difference	Present: 70 (57.9%); Absent: 51 (42.1%)
Left Index-Toe Difference	Present: 74 (61.2%); Absent: 47 (38.8%)
Right Toe Elevation Method	Present: 67 (55.4%); Absent: 54 (44.6%)
Left Toe Elevation Method	Present: 77 (63.6%); Absent: 44 (36.4%)
D. ABI Measurements	
Right ABI (Mean ±SD)	0.76 ±0.16
Left ABI (Mean ±SD)	0.77 ±0.15
Right ABI PVD	Present: 105 (86.8%); Absent: 16 (13.2%)
Left ABI PVD	Present: 108 (89.3%); Absent: 13 (10.7%)
E. Diagnostic Performance	
Right Index-Toe Difference vs ABI	TP: 42; FP: 28; TN: 26; FN: 25; Sensitivity: 62.68%; Specificity: 48.14%; PPV: 60.00%; NPV: 50.98%; Accuracy: 56.19%
Left Index-Toe Difference vs ABI	TP: 49; FP: 25; TN: 19; FN: 28; Sensitivity: 63.63%; Specificity: 43.18%; PPV: 66.21%; NPV: 40.42%; Accuracy: 56.19%
Right Toe Elevation vs ABI	TP: 58; FP: 47; TN: 7; FN: 9; Sensitivity: 86.56%; Specificity: 12.96%; PPV: 55.23%; NPV: 43.75%; Accuracy: 53.71%
Left Toe Elevation vs ABI	TP: 71; FP: 37; TN: 7; FN: 6; Sensitivity: 92.20%; Specificity: 15.90%; PPV: 65.74%; NPV: 53.84%; Accuracy: 64.46%
Right ABI vs Left ABI	TP: 103; FP: 2; TN: 11; FN: 5; Sensitivity: 95.37%; Specificity: 84.61%; PPV: 98.09%; NPV: 68.75%; Accuracy: 94.21%
